# Supplementary material for: Machine learning for laser-induced electron diffraction imaging of molecular structures
Source: Commun Chem. 2021 Nov 9;4:154. doi: 10.1038/s42004-021-00594-z (PMC9814146; doi:10.1038/s42004-021-00594-z)
Supplement: Supplementary file 1 — Supplementary information. [file 42004_2021_594_MOESM1_ESM.pdf]

Supplemental information - Machine learning for laser-induced electron diffraction imaging of molecular structures

Xinyao Liu[1], Kasra Amini[1], Aurelien Sanchez[1], Blanca Belsa[1], Tobias Steinle[1], Jens Biegert[1,2,†]

[1]*ICFO - Institut de Ciencies Fotoniques, The Barcelona Institute of Science and Technology, 08860 Castelldefels (Barcelona), Spain.*
[2]*ICREA, Pg. Lluís Companys 23, 08010 Barcelona, Spain.*

[†]To whom correspondence should be addressed to. Email: jens.biegert@icfo.eu.

Supplementary Note 1 – Database of molecular structures

We generated a database of C_2H_2 molecular structures that spanned C-C (C-H) internuclear distance between 0.8 Å – 2.0 Å (0.0 Å - 2.6 Å). A database for CS_2 was also generated spanning the C-S internuclear distance between 1.3 Å to 2.5 Å and the SCS bond angle from 180° to 70°. For C_2H_2, the molecule is varied along its C-C and C-H bond lengths in cartesian coordinates. Whilst for CS_2, the C-S bond length and the SCS bond angle are varied in the polar coordinate system where we are more sensitive to bond angle changes that have been previously reported[20].

Supplementary Note 2 – Normalization of difference interference signals for different mole cular structures

In the generated database, each molecular structure has a corresponding two-dimensional differential cross-section (2D-DCS) that contains the molecular interference signal which was calculated using the independent atom model (IAM)[1]. Each individual 2D-DCS is normalized between 0-1 by dividing by its corresponding maximum signal. The 2D-DCS of the equilibrium molecular structure was calculated using the IAM and also normalized between 0-1 by dividing by its maximum signal. The 2D-DCS of the equilibrium structure was then subtracted away from each individual 2D-DCS in the database to obtain the normalized difference 2D-DCS interference signal that is normalized between -1 and +1. This is then used as the training dataset to train our machine learning algorithm. FigS1. c-d show the 2D-DCS maps for C_2H_2 and CS_2, respectively, which visually look similar to one another. We enhance the subtle differences that exist in the 2D-DCS maps for the different molecular structures by subtracting from all the maps the calculated 2D-DCS corresponding to the equilibrium molecular structure. This leads to clearly visible fringe patterns in the resulting difference maps for both molecules, as shown in FigS1. e and f. These fringe patterns vary upon changing the molecular structure, making it easier for the neural network to find the relationship between the 2D-DCS maps and the molecular structures.

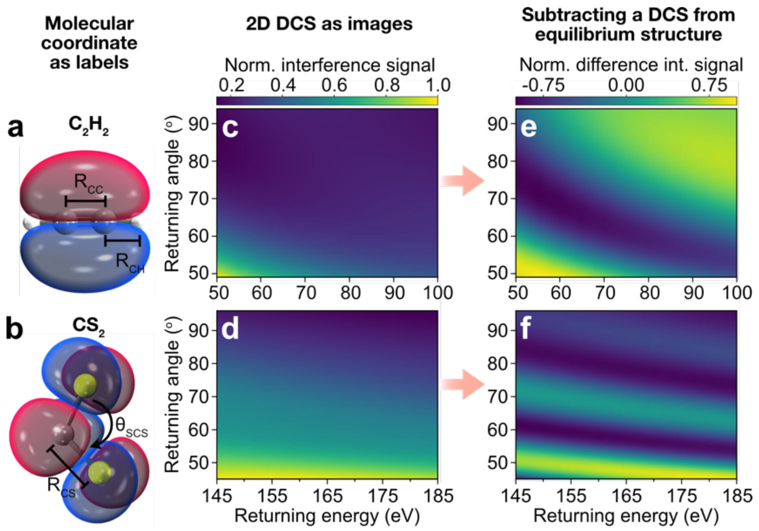

Fig. S1 Elastic scattering of molecules as the input of ML model. The molecular coordinates used as labels for C_2H_2 **(a)** and CS_2 **(b)** molecules in cartesian and polar coordinates, respectively. **c-d** Normalized simulated 2D-DCS maps calculated with the IAM for a single structure of C_2H_2 (c) and CS_2 (d), respectively. **e-f** Difference simulated 2D-DCS map for C_2H_2 (e) and CS_2 (f) calculated from subtracting the calculated 2D-DCS of the equilibrium molecular structure[2,3] from the absolute 2D-DCS maps in (c)-(d). Fringe patterns become visible in the subtracted DCS maps which enhance the difference in the elastic scattering signal for the different molecular structures.

After training the model, we use our experimental data as an input. We first normalize the experimental 2D-DCS between 0-1, and then subtract the normalized 2D-DCS of the equilibrium structure to obtain the normalized difference 2D-DCS for the experimental 2D-DCS which is also normalized between -1 and +1. This is then used as an input for our machine learning model which subsequently generates the predicted molecular structure. We implement this procedure for both experimental and IAM-calculated 2D-DCS data because these two sets of data possess values spanning different orders-of-magnitude which would otherwise be difficult to subtract the calculated DCSs from the measured DCS. Hence, the above-mentioned normalization procedure is required.

Supplementary Note 3 - The beta fitting factor in QRS-LIED

To scale the measured 2D-DCS data to the IAM-calculated 2D-DCS data (see Equation (1a)) in QRS-LIED, a beta fitting factor must be used (see Equation 1b)) since both data possess DCS values of different orders-of-magnitudes.

$$\mathrm{MCF_{theory}}(k_r, \theta_r) = \frac{\sigma_{tot} - \sigma_{atom}}{\sigma_{atom}} \tag{1a}$$

$$\mathrm{MCF_{exp}}(k_r, \theta_r) = \frac{\sigma_{exp,tot} - \sigma_{atom}}{\sigma_{atom}} = \frac{\beta\sigma_{exp} - \sigma_{atom}}{\sigma_{atom}} \tag{1b}$$

This beta fitting factor can be avoided using machine learning (ML) together with the procedure outlined in sub-section II, making the ML approach more robust.

Supplementary Note 4 – Correlation map

A constant offset factor is applied to the experimental DCS due to the relatively lower counts of the experimental DCS in the high return energy range. Specifically, after normalising the DCS of the experimental and simulated data, we add an offset value of 0.07 to the normalised experimental DCS, as given by Equation 2.

$$DCS_{sim} <-> DCS_{exp} + offset \qquad (2)$$

Supplementary Note 5 - Error propagation

A. Model error

The model error originates from the mean absolute error (MAE) between the desired value and calculated value. Here, we use the test dataset to visualize the error for the molecular structure (e.g. internuclear distances and bond angle). The test set contains thousands of pairs, where a pair corresponds to the molecular structure parameter (e.g. internuclear distance) and their corresponding theoretical IAM-calculated 2D-DCS. The theoretical 2D-DCS is used as an input for the ML model which subsequently generates the calculated predicted structure. Taking the difference between this calculated predicted structure and the molecular structure in our database gives the error of our ML model. Taking the average of the absolute difference between the input structure and calculated structure for all molecular structures in the test dataset provides the error of the structures from the model (Equation (3a)), called the MAE.

$$Error_{Model} = averge|desired\ value - calculated\ value| \qquad (3a)$$

B. Experimental error

The experimental error is obtained by using the experimental DCS as an input for our ML model to subsequently generate a predicted molecular structure. The Poisson error is considered since our experimental data obey a Poisson distribution, and our experimental error is a function in the variance of the Poisson distribution given by DCS-√DCS to DCS+√DCS. Taking this range into account as an input, this will give us different predicted molecular structures respectively, where the shift between different predicted molecular structures contributes to our experimental error as shown in Equation 3b.

$$Error_{exp} = \left|f_{(DCS+\sqrt{DCS})} - f_{(DCS)}\right| + \left|f_{(DCS-\sqrt{DCS})} - f_{DCS}\right| \qquad (3b)$$

Supplementary Note 6 – Details of the fully connected neural network

We present the calculation process during the fully connected neural network, ω shown as the weights of each neuron, b present bias value in each layer. Here, we present the computation process for L_{th} layer, assuming in the L_{th} layer consist of k neurons, for layer $L\text{-}1_{th}$ it contain of n neurons, matrix of a^{L-1} as input value in the L_{th} layer, output matrix a^{L} calculated by firstly multiply the matrix of weight element and add bias and then applying an

active function g() which normally a nonlinear function, in equation(4). In general, the computational process for each layer could be described as firstly applied by a linear function for input value then combine a non-linear function. In the meantime, the value a^L matrix is also the input value of next ($L+1_{th}$) layer. The computational process is repeated from first layers until final output layer.

$$\begin{bmatrix} \boldsymbol{a_0^L} \\ \vdots \\ \boldsymbol{a_k^L} \end{bmatrix} = \boldsymbol{g}\left(\begin{bmatrix} \omega_{\boldsymbol{0,0}} & \cdots & \omega_{\boldsymbol{0,n}} \\ \vdots & \ddots & \vdots \\ \omega_{\boldsymbol{k,0}} & \cdots & \omega_{\boldsymbol{k,n}} \end{bmatrix} \begin{bmatrix} \boldsymbol{a_0^{L-1}} \\ \vdots \\ \boldsymbol{a_n^{L-1}} \end{bmatrix} + \begin{bmatrix} \boldsymbol{b_0} \\ \vdots \\ \boldsymbol{b_n} \end{bmatrix} \right) \tag{4}$$

Supplementary References

1.Lin, C. D., Le, A.-T., Chen, Z., Morishita, T. & Lucchese, R. Strong-field rescattering physics—self-imaging of a molecule by its own electrons. *J. Phys. B* **43**, 122001 (2010).
2**.** Herzberg, G. *Molecular Spectra and Molecular Structure III: Electronic Spectra and Electronic Structure of Polyatomic Molecules* (D. Van Nostrand, New York 1966).
3. Dalton, D. R. *Foundations of Organic Chemistry: Unity and Diversity of Structures, Pathways, and Reactions* (John Wiley & Sons, 2020).
